# Hypoxia-inducible factor prolyl hydroxylase inhibitor-induced thrombosis leading to transcatheter aortic valve dysfunction: a case report

**DOI:** 10.1093/ehjcr/ytae658

**Published:** 2024-12-11

**Authors:** Akihiro Ikuta, Syunsuke Matsushita, Kazushige Kadota, Tatsuhiko Komiya, Yasushi Fuku

**Affiliations:** Department of Cardiovascular Medicine, Kurashiki Central Hospital, Kurashiki, Japan; Department of Cardiovascular Medicine, Kurashiki Central Hospital, Kurashiki, Japan; Department of Cardiovascular Medicine, Kurashiki Central Hospital, Kurashiki, Japan; Department of Cardiovascular Surgery, Kurashiki Central Hospital, Kurashiki, Japan; Department of Cardiovascular Medicine, Kurashiki Central Hospital, Kurashiki, Japan

**Keywords:** TAVR, Valve thrombosis, HIF-PH inhibitor, CKD, Anaemia, Case report

## Abstract

**Background:**

Transcatheter aortic valve replacement (TAVR) is a well-established treatment option for patients with severe aortic valve stenosis; however, clinical valve thrombosis is a major challenge.

**Case summary:**

A 92-year-old woman underwent TAVR for severe aortic stenosis. One month later, the patient developed acute heart failure. As the progression of anaemia due to renal anaemia seemed to cause acute heart failure exacerbation, we started an oral hypoxia-inducible factor prolyl hydroxylase (HIF-PH) inhibitor. After 2 weeks, the patient redeveloped shortness of breath. Transthoracic echocardiography revealed that the mean aortic valve pressure gradient (Δ*P*) increased from 9 to 54 mmHg, and the aortic valve area decreased from 1.93 to 0.86 cm^2^. Blood work revealed a markedly elevated haemoglobin level from 8.0 to 13.2 g/dL, and transoesophageal echocardiography revealed markedly decreased left coronary and non-coronary cusp mobility. We diagnosed that the rapid increase in the haemoglobin level caused by the HIF-PH inhibitor was related to valve thrombosis and bioprosthetic dysfunction of the transcatheter aortic valve. The HIF-PH inhibitor was discontinued, and anticoagulation therapy was started. Transthoracic echocardiography at 16 days later revealed that the mean aortic valve Δ*P* improved by 15 mmHg, and the subjective symptoms resolved.

**Discussion:**

This is the first report on a successful treatment of TAVR thrombosis formation associated with HIF-PH inhibitor use. When treating renal anaemia in patients undergoing TAVR, care should be taken to avoid rapid anaemia resolution and valve thrombosis development.

**Learning points**

- To understand rapid anaemia correction using hypoxia-inducible factor prolyl hydroxylase inhibitors can lead to thrombus formation in the transcatheter aortic valve replacement (TAVR) valve and result in bioprosthetic valve dysfunction.
- To learn how to treat a rapidly progressing thrombus formation in the TAVR valve.

## Introduction

Transcatheter aortic valve replacement (TAVR) is a well-established treatment option for patients with severe aortic valve stenosis. Clinical valve thrombosis after TAVR is relatively rare (0.6–2.6%), but this complication is a significant problem with TAVR.^1,2^ Oral anticoagulation therapy can resolve thrombosis, elevated aortic valve pressure gradient (Δ*P*), and clinical symptoms; however, the diagnosis and treatment of clinical valve thrombosis remains unclear.^2^ This is the first case report on a successfully treated bioprosthetic valve dysfunction due to a rapidly developing drug-induced valve thrombosis.

## Summary figure

**Figure:**
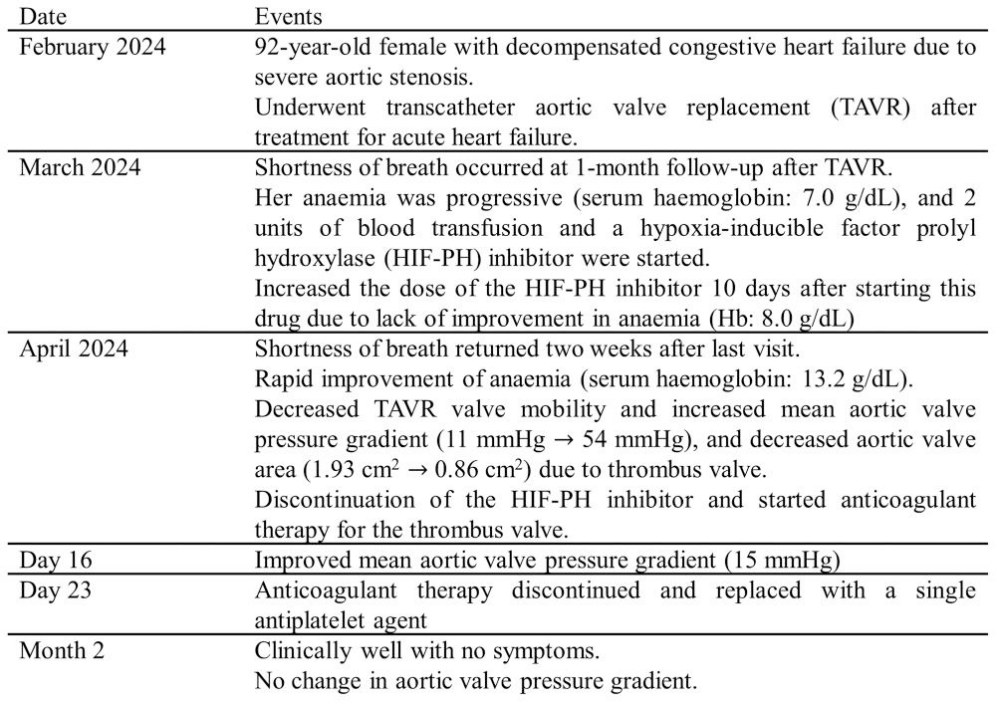


## Case presentation

**Figure 1:**
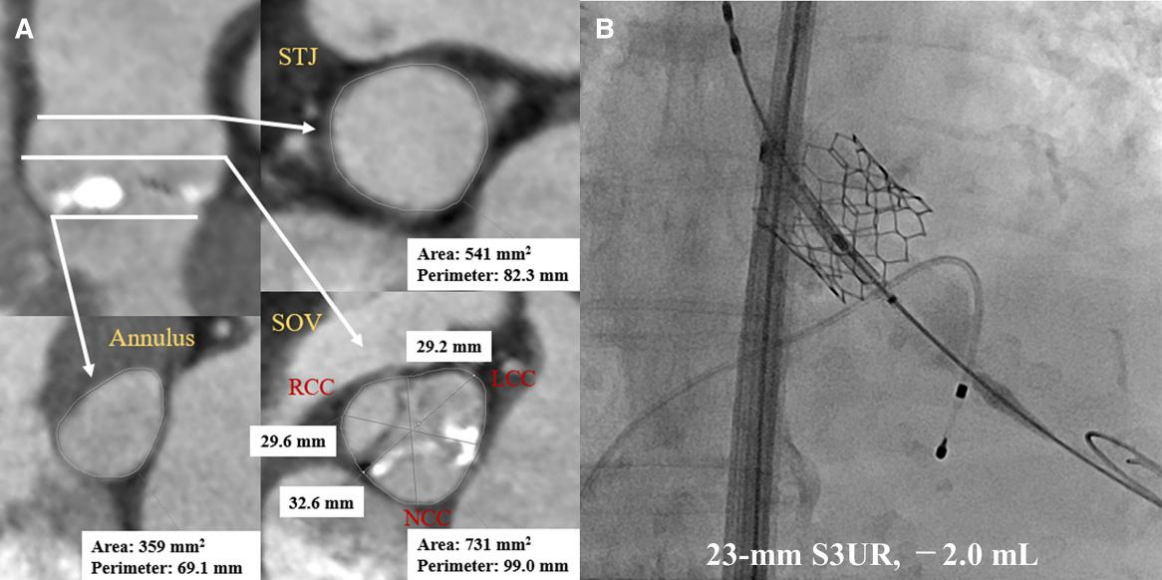
Images of the patient. (*A*) Preprocedural findings on contrast-enhanced computed tomography. Aortic annular area and perimeter are 359 mm^2^ and 69.1 mm, respectively. (*B*) The implanted 23 mm Sapien3 Ultra Resilia transcatheter heart valve with a 2 mL underfilling volume.

A 92-year-old Japanese woman with acute heart failure was presented to our institution. On admission, her consciousness was clear, blood pressure was 169/50 mmHg, heart rate was regular at 75 beats/min, body temperature was 36.7°C, and oxygen saturation was 95% on room air. Physical examination revealed mild oedema of the lower legs. Her heart failure improved with conservative treatment. However, transthoracic echocardiography (TTE) revealed a reduced left ventricular ejection fraction of 49%, severe aortic stenosis with an aortic valve area of 0.69 cm^2^ and a mean aortic valve gradient of 48 mmHg. Blood work showed an estimated glomerular filtration rate of 14.2 mL/min/1.73 m^2^ (reference: <60 mL/min/1.73 m^2^) and a haemoglobin level of 9.2 g/dL (reference: 11.6–14.8 g/dL). Contrast-enhanced computed tomography (CT) angiography revealed that the annular perimeter and area were 69.1 mm and 359 mm^2^, respectively (*Figure 1A*). The patient had a history of interstitial pneumonia, chronic kidney disease (Grade 5), hypertension, and hyperuricaemia; she was on medication for hypertension and hyperuricaemia and had no history of thrombotic events. With a Society of Thoracic Surgeons predicted risk of mortality score of 42.8% and the patient’s wishes, the local heart team deemed the patient ineligible for surgery and consensus was reached for TAVR.

**Figure 2:**
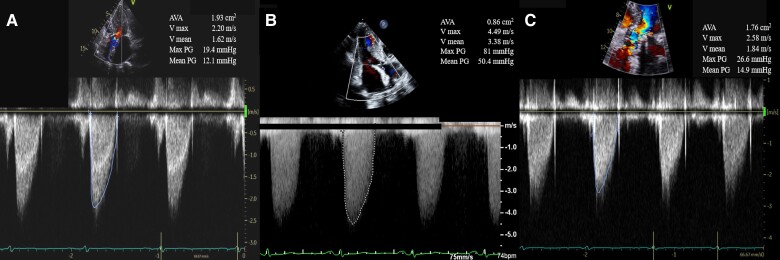
Changes in transthoracic echocardiography. (*A*) Predischarge transthoracic echocardiography findings (4 days following transcatheter aortic valve replacement). (*B*) Transthoracic echocardiography findings at exacerbation (54 days following transcatheter aortic valve replacement). (*C*) Transthoracic echocardiography findings after improvement (70 days following transcatheter aortic valve replacement).

A 23 mm Sapien3 Ultra Resilia transcatheter heart valve (Edwards Lifesciences) was successfully implanted under local anaesthesia (*Figure 1B*), with an initial aortic Δ*P* of 9 mmHg and minimal paravalvular leakage (PVL). However, predischarge evaluation showed a mean aortic Δ*P* of 12 mmHg, with no changes in PVL (*Figure 2A*). At discharge, she was maintained on amlodipine 5 mg/day, febuxostat 10 mg/day, azosemide 60 mg/day, tolvaptan 7.5 mg/day, and aspirin 100 mg/day. At the 1-month follow-up, the patient developed shortness of breath on exertion. Transthoracic echocardiography revealed a mean aortic Δ*P* of 11 mmHg and PVL resolution; however, pulmonary hypertension exacerbation was observed. Blood work showed that the haemoglobin level decreased by 7.0 g/dL and a high ferritin level of 438 ng/mL (reference: 12–60 ng/mL). No obvious bleeding was noted on examination, and the patient was diagnosed with worsening heart failure due to progressive renal anaemia. Two units of red blood cells were transfused, diuretics were increased and an oral hypoxia-inducible factor prolyl hydroxylase (HIF-PH) inhibitor, roxadustat, was started at the usual initial dose of 50 mg three times/week. Blood work on the day after the transfusion showed an increased haemoglobin level of 7.9 g/dL, and the heart failure rapidly improved thereafter. However, blood work at 10 days later showed little improvement, with a haemoglobin level of 8.0 g/dL. Therefore, although earlier than usual, the dose was increased to roxadustat 100 mg three times/week, and the patient was discharged from hospital.

**Figure 3:**
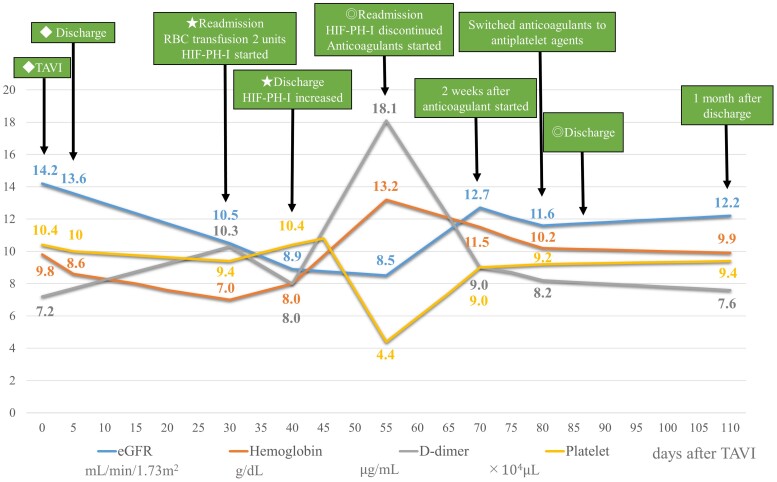
Changes in blood work results.

At the 2-week follow-up, the patient redeveloped shortness of breath. Transthoracic echocardiography revealed no pulmonary hypertension; however, the mean aortic valve Δ*P* increased to 54 mmHg, and the aortic valve area decreased to 0.86 cm^2^ (*Figure 2B*). Blood work revealed a markedly elevated haemoglobin level of 13.2 g/dL, a high D-dimer level of 18.1 μg/mL (reference: <1.0 μg/mL) and a decreased platelet count of 4.4 × 10^4^ μL (reference: 160 × 10^9^–360 × 10^9^/L) (*Figure 3*). Furthermore, C-reactive protein level did not increase. However, the patient’s severe chronic kidney disease had worsened before the TAVR procedure, making contrast-enhanced CT angiography difficult; therefore, transoesophageal echocardiography was performed, which showed markedly reduced left coronary and non-coronary cusp mobility (see Supplementary material online, *Videos S1* and *S2*). Based on these results, we diagnosed that the rapid increase in haemoglobin levels due to the use of HIF-PH inhibitors could lead to exacerbation of valve thrombus and cause bioprosthetic dysfunction of the transcatheter aortic valve.

The HIF-PH inhibitor was discontinued, and warfarin and heparin were started as anticoagulants. Four days after warfarin initiation, the patient reached the therapeutic range (the INR around 2.0), and heparin was discontinued. Subsequently, the D-dimer level gradually decreased with treatment (after the 1-week treatment, 12.9 μg/mL; after the 2-week treatment, 9.0  μ g/mL). Transthoracic echocardiography at 16 days later revealed that the left and non-coronary cusp mobility improved, the mean aortic valve Δ*P* improved by 15 mmHg (*Figure 2C*, Supplementary material online, *Video S3*), and the subjective symptoms resolved. As the thrombogenicity had resolved and the bleeding risk was high with a HAS-BRED score of 3, warfarin was discontinued and the patient was switched to a single antiplatelet agent 7 days after the last TTE.

The patient presented for a 1-month follow-up with continued improvement in symptoms. She was treated with a single antiplatelet agent without restarting anticoagulation, and the mean aortic valve Δ*P* remained at 16 mmHg without worsening. The D-dimer level was also not elevated at 7.6  μg/mL (*Figure 3*).

## Discussion

This is the first report of a successful treatment of TAVR thrombosis formation associated with HIF-PH inhibitor use. Most patients with valve thrombus presented with progressive dyspnoea, and the most frequently observed echocardiographic feature was increasing transvalvular gradients.^3^ A 3-month anticoagulation therapy is recommended for valve thrombosis in cases that required treatment; however, the risk factors for valve thrombosis and the appropriate treatment duration are not well elucidated.^4^ Furthermore, anticoagulation therapy is a major risk factor for bleeding in older adults.^5^ In this case, the patient was at a high risk of bleeding; at 16 days after starting anticoagulation, the mean aortic valve Δ*P* improved, and one of the causes of the valve thrombosis was eliminated. Therefore, we switched anticoagulation to an antiplatelet therapy within 1 month; the patient showed a favourable treatment course.

Approximately 50% of the patients undergoing TAVR have anaemia, and renal anaemia is associated with patients with anaemia who underwent TAVR.^6,7^ Furthermore, when patients with anaemia who underwent TAVR improved their anaemia, the incidence of rehospitalization within 1 year following TAVR decreased.^8^ In a previous report, renal anaemia was safely treated by starting an HIF-PH inhibitor, increasing the dose after 10 weeks while monitoring haemoglobin changes, and increasing the haemoglobin level by 1.0 g/dL over a 6-month period.^9^ In this case, the dose of the HIF-PH inhibitor was increased after 10 days of administration, and 2 weeks later, the patient developed thrombosis due to a 5.2 g/dL increase in haemoglobin. Although previous studies may differ from this case as the patient was receiving anticoagulants, other studies have reported that rapid haemoglobin elevation with HIF-PH inhibitors is a risk factor for thrombus formation.^10^ Thus, although HIF-PH inhibitors may be effective for improving renal anaemia in patients undergoing TAVR, care should be taken not to cause a rapid increase in haemoglobin levels.

## Conclusion

The rapid increase in the haemoglobin level with HIF-PH inhibitor use was related to the rapid thrombus formation and bioprosthetic valve dysfunction of the TAVR valve. Patients undergoing TAVR are frequently complicated by renal failure and anaemia, and attention should be paid to anaemia treatment.

## Supplementary Material

ytae658_Supplementary_Data

## Data Availability

Anonymized data underlying this article will be shared on reasonable request to the corresponding author.

## Lead author biography

Dr Akihiro Ikuta earned his MD from Shiga University of Medical Science in 2013 and is currently a physician at the Department of Cardiology of Kurashiki Central Hospital. His research interests include severe aortic stenosis and kidney diseases to improve their long-term outcomes.

## Supplementary material

**Consent:** The authors confirm that patient consent was obtained in accordance with COPE guidelines. The authors certify that they have obtained the patient’s consent to the publication of information about her in a journal or thesis.
